# The physical and functional borders of transit peptide-like sequences in secondary endosymbionts

**DOI:** 10.1186/1471-2229-10-223

**Published:** 2010-10-19

**Authors:** Gregor Felsner, Maik S Sommer, Uwe G Maier

**Affiliations:** 1 Laboratory for Cell Biology, Philipps University Marburg, Karl-von-Frisch Str.8, D-35032 Marburg, Germany; 2 Department of Molecular Cell Biology of Plants, Goethe-University of Frankfurt, Max-von-Laue Str. 8, D-60438 Frankfurt, Germany

## Abstract

**Background:**

Plastids rely on protein supply by their host cells. In plastids surrounded by two membranes (primary plastids) targeting of these proteins is facilitated by an N-terminal targeting signal, the transit peptide. In secondary plastids (surrounded by three or four membranes), transit peptide-like regions are an essential part of a bipartite topogenic signal sequence (BTS), and generally found adjacent to a N-terminally located signal peptide of the plastid pre-proteins. As in primary plastids, for which no wealth of functional information about transit peptide features exists, the transit peptide-like regions used for import into secondary ones show some common features only, which are also poorly characterized.

**Results:**

We modified the BTS (in the transit peptide-like region) of the plastid precursor fucoxanthin-chlorophyll a/c binding protein D (FcpD) fused to GFP as model substrate for the characterization of pre-protein import into the secondary plastids of diatoms. Thereby we show that (*i*) pre-protein import is highly charge dependent. Positive net charge is necessary for transport across the plastid envelope, but not across the periplastid membrane. Acidic net charge perturbs pre-protein import within the ER. Moreover, we show that (*ii*) the mature domain of the pre-protein can provide intrinsic transit peptide functions.

**Conclusions:**

Our results indicate important characteristics of targeting signals of proteins imported into secondary plastids surrounded by four membranes. In addition, we show a self-targeting mechanism, in which the mature protein domain contributes to the transit peptide function. Thus, this phenomenon lowers the demand for pre-sequences evolved during the course of endosymbiosis.

## Background

Primary plastids are organelles of endosymbiontic origin [e.g. 1, 2]. In the course of the transition from an (endo-)symbiont to an organelle, most of its genes were either lost or, to a higher degree, transferred into the cell nucleus [e.g. 3, 4, 5]. Hence, most of the plastid proteome is encoded in the nucleus of the host cell, implying that the encoded proteins must be transported post-translationally across the two envelope membranes into the plastid lumen. For accurate trafficking, nearly all nuclear-encoded plastid proteins are equipped with a characteristic N-terminal topogenic signal sequence, the transit peptide [6]. This targeting information is necessary and sufficient for plastid import and interacts with translocons of the outer/inner envelope membrane of chloroplasts [TOC and TIC; recently reviewed in 7]. Interestingly, surveys of transit peptides indicate no strict consensus sequence [8] but some common features such as a positive net charge, elevated levels of hydroxylated amino acids and binding motifs for molecular chaperones [9 and references therein].

Secondarily evolved organisms such as diatoms, apicomplexa or cryptophytes harbour plastids surrounded by two additional membranes [10,11]. Genomic analyzes indicated a common set of nuclear-encoded proteins with a plastid destination as in primary plastids [4]. In contrast to the primary plastids, proteins here are equipped with a bipartite topogenic signal sequence (BTS), consisting of a classical ER-like signal peptide (SP) followed by a transit peptide-like sequence (TP) [2,12,13]. This transit peptide-like sequence is - as in archaeplastida - indispensable for plastid import as shown by *in vivo *experiments on apicomplexa and diatoms [5,14,15]. Recently, Tonkin et al. [16] demonstrated that even randomly picked sequences, which follow the basic rules for transit peptides (see above), could function as targeting sequences in apicomplexa, indicating a low complexity of transit peptides. However, in diatoms and cryptophytes, at least one major difference to the apicomplexan transit peptide composition exists, which is the presence of a highly conserved aromatic amino acid at position +1 of the TP crucial for plastid protein import [5,15,17]. The TPs of apicomplexa are not as heavily dependent on the phenylalanine as diatoms and cryptophytes [18].

**Figure 1:**
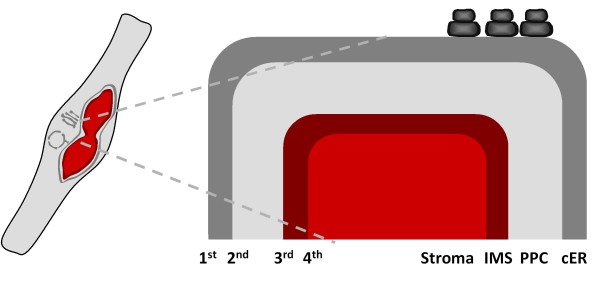
**Schematic depiction of the plastid architecture of *P. tricornutum***. The complex plastid is surrounded by four membranes (counted from outside to inside) with the outermost one being continuous with the endoplasmic reticulum. The cER is studded with ribosomes facilitating co-translational import of plastid precursors across the 1^st ^membrane into the ER lumen. The candidates for translocons of the subsequent membranes (not shown) of secondary plastids with red algal ancestry have been elucidated recently [see 30, 31, 32, 33, 38, 40, 41, 42]. cER, chloroplast endoplasmic reticulum; PPC, periplastid compartment; IMS, intermembrane space.

In order to investigate further features in secondary transit peptide-like regions, we comprehensively studied in the diatom *Phaeodactylum tricornutum *the targeting behaviour of GFP fused to the BTS of the fucoxanthin-chlorophyll a/c binding protein D (FcpD) with modifications in the transit peptide-like region. *P. tricornutum *is the most appropriate system for such studies, since - contrary to apicomplexan parasites like *Plasmodium falciparum *- intermediates that are either transported across one of the four surrounding membranes into the chloroplast ER (cER) only or transported across two into the periplastid compartment (PPC) (Figure 1) [1] can be easily monitored and discriminated from completed import (across all four envelope membranes). Our studies confirmed that (*i*) a positive net charge is critical for protein transport across the innermost two plastid membranes (in case of an aromatic amino acid at the +1 position of the TP), whereas transport across the second outermost membrane obviously is not governed in that way. Here, negative charges hinder a membrane passage. Moreover, we demonstrate that (*ii*) the N-terminus of the mature protein can contribute to the functional necessities of the transit peptide-like sequence. Thus, our findings may additionally indicate how transit peptide-like regions have evolved during the course of evolution.

## Results

### Protein import is charge-dependent

Because transit peptides of land plants possess a positive net charge crucial for proper import into chloroplasts [9], we first tested the localization of GFP fused to the BTS of the fucoxanthin-chlorophyll a/c binding protein D (FcpD) of the diatom *Phaeodactylum tricornutum *with respect to the transit peptide (TP) charge. We chose FcpD (NCBI accession number Z24768) as our model precursor, since its 24 amino acids (aa) long BTS displays all features of a typical diatom plastid pre-protein [e.g. ref. 19 and references therein]. The signal peptide (M1-A15) of the BTS is predicted by SignalP to be cleaved after position 15, giving way to the typically short transit peptide-like region [8] with the conserved phenylalanine (F16) at +1. The transit peptide (9 aa long (F16-R24, predicted by ChloroP) is as expected enriched in hydroxylated aa and alanine, and contains two positively charged residues, K20 and R24. Acidic residues are absent. We draw the line between BTS and mature domain of the protein between aa N30 and M31, since the conserved region of the chlorophyll a-b binding protein domain starts at F33. Hence, we replaced the M31 and the following aa of the mature domain with our reporter - eGFP - turning aa N30 to T.

**Figure 2:**
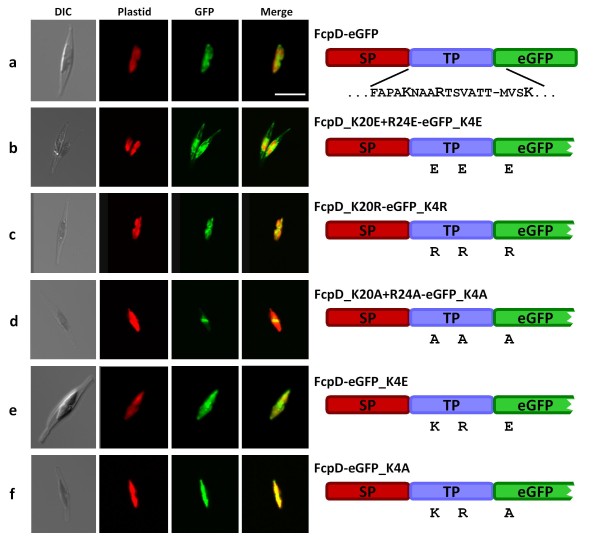
**Import of FcpD-GFP is charge-dependent**. GFP is directed to the stroma by means of the FcpD-BTS of *P. tricornutum *irrespective of the particular positively charged residue (a, c, e, f). Negative charge of the precursor N-terminus impedes import within the ER (b). Neutralising the charges leads to GFP import into the PPC (d). In the scheme of the investigated precursors (on the right side), the amino acid sequence of the FcpD-transit peptide and the very N-terminus of GFP is denoted with charged residues in bold capitals in the topmost line. In further lines, only the charged amino acids at the respective position are indicated. Signal peptide in red (SP), transit peptide in blue (TP), GFP in green (eGFP). DIC = differential interference contrast, Plastid = chlorophyll autofluorescence, GFP = GFP fluorescence, Merge = merged plastid and GFP images. Scale bar = 10 μm.

At first, we fused the wild type BTS to GFP, resulting in an expected localization of GFP in the plastid stroma (Table 1, Figure 2a). Next, we substituted the two positively charged amino acids of the TP and - because of its shortness, to exclude an unintentional influence, also the positive charge of the GFP N-terminus (lysine K4) with glutamate by site-directed mutagenesis. Logically consistent with the plant model, substituting these residues with negatively charged amino acids should "trap" GFP within the upstream compartments (cER/PPC). Expectedly, the reversal of the net charge prevents GFP from being translocated successfully into the stroma to a substantial amount, as denoted by predominant fluorescence in the ER (Figure 2b). To demonstrate that the general net positive charge of a transit peptide but not a specific aa residue at the N-terminus of plastid-destined precursor is crucial for import, we replaced the glutamate residues with arginine residues in a second round of mutagenesis. Here, GFP localizes to the plastid again (Figure 2c). Because the analysis of the dependence on positive charges might lead to erroneous interpretations by the introduction of negative charges we decided to neutralize the pre-protein at the respective amino acid positions by substitution with alanines. Thereby the fusion protein gets imported across the second outermost membrane into the PPC (Figure 2d). Finally, by fusing the unmodified BTS to a mutated GFP species with a glutamate or alanine at position 4 (K4E and K4A, respectively) instead of the genuine lysine, the BTS itself sufficed for correct protein import into the stroma (Figure 2e and 2f, respectively). Additionally, these experiments show that the modified version of GFP still shows fluorescence, functionally identical to the wild type version.

**Table 1:** Constructs investigated in this study

Construct name	**Protein sequence**^**a**^	**Net charge**^**b**^	**Localization**^**c**^
FcpD-eGFP	...FAPA**K**NAA**R**TSVATT-MVS**K**...	+3	S
FcpD-eGFP_K4E	...FAPA**K**NAA**R**TSVATT-MVS**E**...	+1	S
FcpD-eGFP_K4A	...FAPA**K**NAA**R**TSVATT-MVS**A**...	+2	S
FcpD_K20E+R24E-eGFP_K4E	...FAPA**E**NAA**E**TSVATT-MVS**E**...	-3	ER
FcpD_K20R-eGFP_K4R	...FAPA**R**NAA**R**TSVATT-MVS**R**...	+3	S
FcpD_K20A+R24A-eGFP_K4A	...FAPA**A**NAA**A**TSVATT-MVS**A**...	0	PPC
FcpD_K20E+R24E-eGFP	...FAPA**E**NAA**E**TSVATT-MVS**K**...	-1	ER/PPC
FcpD_K20E+N21K+R24E-eGFP	...FAPA**EK**AA**E**TSVATT-MVS**K**...	0	PPC
FcpD_K20E+N21K+R24E-eGFP_K4E	...FAPA**EK**AA**E**TSVATT-MVS**E**...	-2	ER
FcpD_K20A+R24A-eGFP	...FAPA**A**NAA**A**TSVATT-MVS**K**...	+1	PPC
FcpD_K20E+N21R+R24E-eGFP_K4R	...FAPA**ER**AA**E**TSVATT-MVS**R**...	0	PPC
FcpD_K20E+N21R+R24E-eGFP_K4E	...FAPA**ER**AA**E**TSVATT-MVS**E**...	-2	ER
FcpD_Δ20-30-eGFP	...FAPA-MVS**K**...	+1	S
FcpD_Δ20-30-eGFP_K4R	...FAPA-MVS**R**...	+1	S
FcpD_Δ20-30-eGFP_K4E	...FAPA-MVS**E**...	-1	ER
FcpD_Δ20-30-eGFP_K4A	...FAPA-MVS**A**...	0	PPC
FcpD_3xSpacer-eGFP	...FAPA**K**NAA**R**TSVATT-[M(GGGGP)_5_]_3_-MVS**K**...	+2	S
FcpD_Δ20-30-Spacer-eGFP	...FAPA-[M(GGGGP)_5_]-MVS**K**...	0	S
FcpD_Δ20-30-2xSpacer-eGFP	...FAPA-[m(GGGGP)_5_]_2_-MVS**K**...	0	S
FcpD_Δ20-30-3xSpacer-eGFP	...FAPA-[M(GGGGP)_5_]_3_-MVS**K**...	0	ER
FcpD_Δ20-30-3xSpacer_G4K-saGFP11	...FAPA-[M(GG**K**GP)-(GGGGP)_4_]_3_-MVS**K**...	+1	ER/S

^a^Sequence of the FcpD transit peptide, of the eGFP N-terminus and, in spacer constructs, of the synthetic spacers separated by hyphens, respectively; charged amino acids (or substituted alanines) in bold; ^b^Calculated from the bold-faced amino acids, in spacer constructs up to the end of the first spacer; ^c^S: stroma; ER: endoplasmic reticulum; PPC: periplastid compartment.

### The mature protein can possess transit peptide function

**Figure 3:**
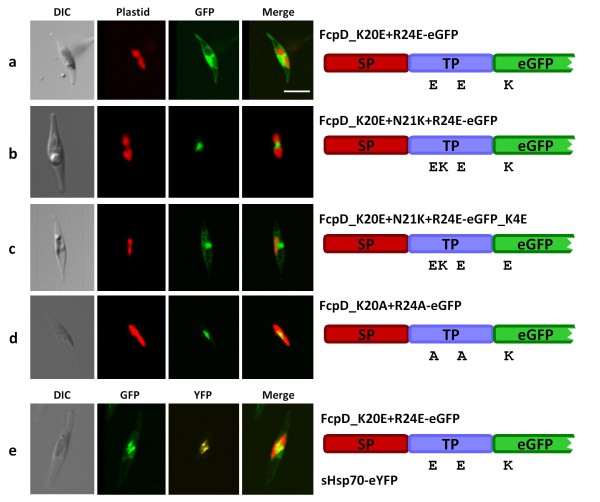
**GFP conveys transit peptide characteristics to a deficient targeting signal**. Wild type GFP is partially transported into the PPC when following a TP with solely negative charges (a, e). Upon introduction of a lysine in the negatively charged TP, GFP carrying a lysine at position four is transported across the PPM (b), whereas GFP with glutamic acid at the N-terminus is not (c). A charge-neutral TP directs wildtype GFP into the PPC (d). Drawing on the right according to figure 1. In the bottom line, colocalization of the FcpD_K20E+R24E-eGFP (construct in topmost line) with a PPC-localized YFP (due to a sHsp70-BTS) is shown. DIC = differential interference contrast, GFP = GFP fluorescence, YFP = YFP fluorescence, Merge = merged images of GFP, YFP and chlorophyll autofluorescence, respectively. Scale bar = 10 μm.

To monitor whether the mature protein can contain topogenic information, we first generated a construct in which a negatively charged transit peptide (K20E, R24E) was fused to wild type GFP. As shown in Figure 3a, fluorescence was predominantly in the ER, but GFP seemed to partially localize to the PPC as well, which was indeed the case as shown by co-localization with PPC-localized eYFP (Figure 3e). Here, eYFP is directed into the PPC by means of a BTS of the PPC marker sHsp70 [20]. By introducing an additional basic aa in between the negatively charged residues (N21K) within the transit peptide (the net charge of the transit peptide and the crucial residue of the GFP equals zero now), it was possible to remediate precursor translocation across the PPM but not across the plastid envelope (as seen by the typical blob-like GFP fluorescence of the PPC [17, 21]; Figure 2b). By modifying this construct by replacing the lysine with a glutamate at position 4 of GFP, ER localization was obtained once again (Figure 3c). The labelled round structure also represents GFP within the nuclear envelope, which is part of the ER (not to confound with blob-like structures, which mark the PPC). Thus, import of the FcpD precursor seems highly dependent on the net charge of the N-terminal extension, and not on a specific aa composition, as analyzed by fusion constructs carrying arginines instead of lysines (data not shown, Table 1). To analyze the contribution of the mature protein that was shown in Figure 3a (FcpD_K20E+R24E-eGFP), we neutralized the TP (K20A, R24A) and fused it to wild type GFP which leads to PPC import of the GFP (Figure 3d). Although the mature protein influences pre-protein targeting when the TP region is negatively charged, the lysine of the GFP does not promote further import stages into the plastid. Altogether, pre-protein import into the PPC seems not strictly dependent on positive charges but these can compensate for present acidic charges.

**Figure 4:**
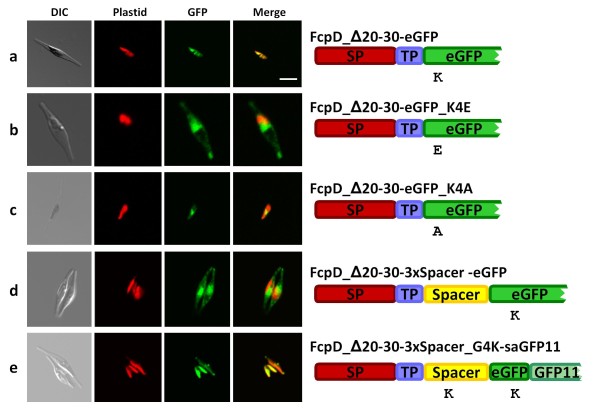
**Import properties of precursors with a truncated transit peptide**. Wild type GFP with lysine at position four is stromally targeted with a truncated FcpD-TP (a), whereas GFP_K4E and GFP_K4A are not (b, c). GFP following spacer sequences is imported into the plastid only if the spacer exhibits positive charge (e), otherwise GFP localizes to the ER (d). Drawing on the right according to figure 1 with the synthetic spacer sequences in yellow (Spacer) representing a threefold incorporation (for spacer sequence see Table 1). Self-assembly GFP fluorescence is shown in the bottom line. Fragment 11 was cloned to the very N-terminus of GFP (see experimental procedures), fragment 1-10 was directed to the stroma by an AtpC-BTS. DIC = differential interference contrast, Plastid = chlorophyll autofluorescence, GFP = GFP fluorescence, Merge = merged plastid and GFP images. Scale bar = 10 μm.

To analyze this charge dependence in more detail, and further discern the influence of the GFP reporter on protein import, we truncated the presequence of the FcpD BTS and deleted the entire charged region of the TP yielding the sequence "FAPA" (ΔK20-T30; with F at position +1). If the conclusions drawn from our earlier observations were correct, this shortened TP would no longer retain its functionality, but however surprisingly, it directs wild type GFP into the stroma (Figure 4a). In contrast, the GFP_K4E - in which the lysine at position 4 was substituted with a glutamate - is not longer stromally targeted (Figure 4b). The influence of the mature protein is reflected, when substituting the lysine of GFP with alanine (K4A). Here GFP gets stuck in the PPC again (Figure 4c). In order to confirm this in a further experimental approach, we introduced a synthetic spacer, lacking charge (which is the important characteristic for this study at hand) with the sequence [M(GGGGP)_5_] between the sequence FAPA and GFP to spatially separate potential targeting information within the GFP from the BTS. Notably, neither the introduction of one nor of two subsequent spacers failed to block pre-protein import, because these precursors were still imported into the plastid (Table 1). Only by inserting a total of three repeats (FAPA-[M(GGGGP)_5_]_3_-GFP; see Table 1) a defect in protein import was observed. Here, the shortened, basic charge lacking presequence did not suffice for stromal protein import, as seen by the GFP fluorescence observed in the ER and the nuclear envelope (Figure 4d). If GFP really provides targeting information itself (due to its lysine at position 4), the introduction of a positive aa into the [M(GGGGP)_5_]_3_ spacer should restore proper GFP targeting into the plastid stroma. Therefore we substituted the third glycine in the spacer sequence with a lysine (FAPA-[M(GG**K**GP)-(GGGGP)_4_]_3_-GFP; see Table 1), which then mimicked the positive charge of the GFP at position 4. As this construct, which can be hardly dissected by microscopic analysis, seemed only to be meagrely imported into the stroma, while most of the fusion protein remained in the ER (data not shown), we chose the self-assembly GFP (saGFP) system [22] to verify protein import into the stroma. Here, GFP is separated into two pieces (GFP1-10 and GFP11), which self-assemble and hence fluoresce when present in the same cellular compartment. For this approach, we directed the saGFP1-10 fragment into the stroma by adding the BTS from the nuclear-encoded plastidic ATPase subunit C (AtpC) of *P. tricornutum *and co-expressed this construct together with the saGFP11 fragment C-terminally fused to the FAPA-[M(GG**K**GP)-(GGGGP)_4_]_3_ construct. By the use of this technology we were therefore able to visualize solely that fraction of precursors which enter the plastid stroma (and not that part of the fusion protein that is stuck in the ER). As presented in Figure 4e, fluorescence was detected exclusively in the stroma as expected, which confirms that indeed a fraction of the fusion protein is successfully imported.

## Discussion

The enslavement and reduction of once free-living cyanobacteria and phototrophic protists to what we today refer to as primary or secondary plastids was accompanied by subsequent cellular and genomic reductions. The plastid genome shrunk enormously by massive gene transfers [and by gene losses; e.g. 23] to the host nucleus. Nonetheless, most of the gene products are still required to maintain plastid function so that the bulk of proteins must be re-imported into the plastid post-translationally. Hence, the development of efficient import machineries for pre-proteins destined for the various sub-compartments of the plastid was doubtlessly indispensable in plastid evolution. Pre-protein import into primary plastids is mediated by an N-terminal targeting signal, the transit peptide [6]. Extensive analyzes of various plant transit peptides reported only some "weak" common features like e.g. a positive net charge [9], which was shown to be essential to successfully cross the plastid envelope. Such features were detected in bioinformatic investigations of transit peptide-like sequences of secondary plastid-bearing organisms as well [19]. To date, protein import into complex plastids can be studied *in vivo *by light-microscopic approaches using fluorescently labelled proteins. However, the exact interpretation of the obtained results is often insufficient because of the resolution limits of microscopes. For instance, evaluation of successful pre-protein import into the four-membrane-bound plastid (apicoplast) of apicomplexan parasites such as *Plasmodium falciparum *is always an all-or-none decision, as one cannot distinguish between the sub-apicoplast compartments. In contrast, the cell size of the diatom *P*. *tricornutum *gives rise to the advantage that not only import into the plastid stroma can be investigated, but also transport across only some of the four surrounding envelope membranes can be tracked [5,15,17]. Thus, application of *P. tricornutum *as a model system for studying pre-protein import into secondary plastids makes such studies more precise.

Like in land plant plastids, transit peptide-like function in *P. tricornutum *relies on the presence of basic/positively charged aa residues [24,25]. This holds true for the BTS of the FcpD precursor fused to GFP as well. Moreover, pivotal for proper transit peptide function is not the particular moiety, but solely the positive charge present (Figure 2c, e, f). In particular, substitution of the charged residues to alanines reveals that, as a first approximation (charge dependent PPM translocation will be discussed below), protein import across the PPM might not be charge dependent, but is across the plastid envelope (cf. Figure 2a and 2d). In addition, the substitution of the basic residues with acidic/negatively charged aa totally impedes import into the stroma (as seen by the ER-localization of the GFP in Figure 2b). A high load of N-terminally located negative charges might be a mechanism to prevent ER-resident proteins from being accidentally PPC-targeted. For example, the ER-residential Hsp70 chaperone BiP and the ER-residential ER quality control protein Calreticulin exhibit four acidic charges in the first six and eleven amino acids, respectively, at the N-terminus of the mature protein.

Secondary plastids of diatoms are surrounded by four membranes. The outermost of these is surmounted by pre-proteins using the SP of the BTS to enter the chloroplast ER lumen [2]. From then on, the TP mediates the passage across the sequent membranes. The lack of a functional TP leads to an arrest of the pre-protein within the ER or the PPC in the diatom (as seen above) and even to its secretion in apicomplexan parasites [24,25], in which the secondary symbiont does not directly reside within a perinuclear ER cisterna [a detailed description of plastid morphology is given in 1]. Hence, the transit peptide facilitates the passage of the pre-protein across the former symbiontic plasma membrane (the periplastid membrane, PPM) before the pre-proteins gain access to the genuine (primary) plastid envelope. The TP of the FcpD BTS, in which the positively charged residues are substituted with glutamate (K20E, R24E see Figure 3 and Table 1), suffices for at least a partial import of GFP into the PPC (Figure 3a, d). Here, the transit peptide net charge is negative (-2 or -1 when K4 of GFP is included). Therefore, the generic plant-like transit peptide characteristics seem to be less important for the passage across the PPM than for the passage across the plastid envelope. We mentioned above that transport across the PPM can take place in a charge independent manner, but as seen in Figure 3a and 3e positive charges can outweigh negative ones. The fact that GFP is only partially imported might be caused by the order and number of the charged aa within the TP. Here, the first two negative charges may somehow shield the basic residue of the GFP and interfere with the translocon in the PPM. This is consistent with the results obtained with a construct, in which an additional lysine (N21K) was introduced between the negatively charged TP (Figure 3b). Here, the GFP was entirely imported into the PPC. However, by fusing this TP to the GFP, in which K4 was substituted with a glutamate (Figure 3c), GFP fluorescence was obtained exclusively in the ER. From these results, the following two essential consequences can be deduced: (*i*) An impact of the reporter GFP on pre-protein import as already shown in Figure 3a becomes evident and (*ii*) import across the PPM can depend on discrete positively charged residues rather than on a global basic net charge of the TP, but not in either case (see Figure 2d). Notably, Tonkin et al. reported that positive aa in the N-termini of apicoplast TPs are essential for proper apicoplast import as well [25]. But due to the different morphology of the apicoplast, pre-protein import is already halted at the stage of apicoplast targeting/recognition.

As outlined before, the TP of the FcpD BTS is compared to plant TP relatively short, and an influence of the mature part of FcpD on pre-protein import was taken into account. To investigate this in more detail, we trimmed the TP such that the four remaining residues of the transit peptide lack any charge. According to our and previous data [outlined in 9], the TP should have been dysfunctional but it was not (Figure 4a). Contrarily, the truncated TP fused to the K4E GFP stopped protein import from proceeding beyond the ER. Interestingly, the construct comprising the shortened TP and the K4A_GFP led to a localization within the periplastidal compartment (Figure 4c). This means that the residual TP aa, which can be hardly termed TP-like anymore, mediates translocation of a further membrane. This result suggests that the TP is not required for the transport across the PPM. As a consequence, one would no longer consider the acquisition of the BTS - and therefore of a TP - of PPC-targeted proteins as critical step for proper targeting. Quite the opposite, it seems that non aromatic or to some level acidic aa within the TP have to be present, to prevent aberrant (stromal) targeting. At present, we cannot define, whether the information for the PPM passage resides within the remainder of the TP (the sequence FAPA) or within the GFP (or a combination of both).

To demonstrate the influence of the GFP lysine in more detail, we introduced a spacer peptide between the truncated TP and wild type GFP (see Figure 4d,e; Table 1). These experiments clearly indicate the assistance of the reporter on pre-protein translocation across the PPM. Surprisingly, only a threefold repeat was able to impede GFP import (Figure 4d). While this manuscript was in revision, Bionda and coworkers published that the critical length requirement of TPs of plant primary plastids is about 60 aa [26]. This is in line with our observations, since a twofold and threefold spacer repeats add up to 52 and 78 aa, respectively. After introducing a lysine into the spacer GFP import was at least partially re-established (Figure 4e). Consistently, the reporter - or to generalize - the mature domain can influence its transport by contributing essential aa for TP functionality. The poor efficiency of stromal import obtained in these experiments is most likely due to the composition of the spacer, whose sequence does not resemble the classical make-up of TPs commonly present in diatoms [15,19]. At the moment, we cannot exactly specify the distance that can be bypassed by positive charges to act on TPs for proper targeting. In the spacer constructs, as mentioned above, a twofold repetition of the synthetic spacer, which totals 52 amino acids, did not block plastid import (Figure 4d). On the other hand, wild type GFP cannot drive plastid import when fused to a neutralized TP (K20A, R24A, see Figure 3d). Supposably, the environment of the charged residue affects the contribution to the targeting process. In any case, these results contradict previous attempts to define the minimal unit of the TP that is indispensable for accurate protein targeting [15,27] and indicate that the physical and functional borders of a TP are not necessarily identical. This means that the acquired N-terminal extension during endosymbiosis, the physical TP, does not contain always all features that are essential for targeting, the functional TP. We have therefore shown that the characteristics of topogenic signals required for pre-protein import into secondary plastids, as inferred from previous studies [5], must be supplemented by our data on TP positive charge presented here. Anyhow, the observed discrepancy of the functional/physical TP border to the mature protein hampers the prediction of (peri-)plastid-directed proteins, as these are commonly identified by characteristics of "self-sufficient" transit peptides, meaning those which contain all topogenic information necessary for import. Potential plastid pre-proteins therefore, if preceded by a rather short BTS or SP only, should be re-examined and the N-terminal portion of the mature domain has to be taken into account, while predicting a (sub-)cellular localization of a protein. On the other hand the *in vivo *localization of a putative plastid protein by genetically fusing a potential BTS to GFP might be misleading.

## Conclusions

Since organellar genes have been transferred to the nuclear genome in secondarily evolved organisms, the acquisition of appropriate targeting signals was a key issue for their accurate re-direction to the plastid sub-compartments. As even random sequences that match only the elementary characteristics of TPs, serve as functional pre-sequences [16], the complexity of a genuine TP seems to be rather low. Therefore the constraints for a DNA sequence to act as a functional transit peptide is diminished to the most basic features. Hence, if (plastid) proteins - as exemplified above - possess intrinsic information for their own (sub-)organellar targeting spontaneous self-targeting might be an evolutionary driving force that facilitated and/or accelerated the successful integration of the transferred genetic material in to the new genetic background. Thus, transit peptides may evolve by exon shuffling from pre-existing plastid-targeted genes [6,16,21] and/or by the help of the here demonstrated self-targeting mechanism, by which the transit peptides are moulded from the N-terminal part of the mature protein by subsequent mutation.

In summary, our results offered new insights into the features of pre-protein targeting signals in secondary plastids. Beyond its requirement for crossing the plastid envelope, discrete positive aa residues can play a role in the import across the PPM. Common transit peptide features are not essential for this import step. In addition, we demonstrated that the mature domain of the protein (here the GFP reporter) facilitated its own translocation, consequently reducing the degree of topogenic capacity necessary in the signal preceding the mature protein. This self-targeting in turn might have enhanced the successful expression of plastid genes targeted to the nucleus during evolution.

Lastly, our results presumably can be generalized for the other plastid-harbouring organisms as well, since *(i) *many factors of the translocation machineries are conserved in archaeplastida [28,29], *(ii) *some of them, as well as the putative import machinery embedded in the PPM, are present in complex plastids and the apicoplast, respectively [30-33], and *(iii) *transit peptides are cross-species functional [e.g. 20, 34, 35, 36, 37].

## Methods

### Cloning

eGFP and eYFP constructs were generated with terminal 5' *Eco*RI and 3' *Hind*III restriction sites. The presequences were linked via *Nco*I sites to GFP and YFP, respectively. DNA fragments were amplified using the PHUSION High-Fidelity DNA polymerase (Finnzymes, Espoo, Finland) or Biotools DNA polymerase (Madrid, Spain). Amino acid substitutions were carried out by PCR-based mutagenesis using primers with respective sequences (see Additional file 1). Presequences and the GFP/YFP-encoding fragment were ligated into the transformation vector pPhaT1 in one step. Prior to the integration of the spacer sequences (provided by GeneArt, Regensburg, Germany), the respective construct was temporarily integrated into pBluescript II KS+. Linearization by restriction with *Nco*I and dephosphorylation of DNA termini by shrimp alkaline phosphatase preceded the addition of the spacers. The small fragment (GFP11) of the self assembly GFP system [22] was introduced 5' via the first *Alu*I restriction site of eGFP and 3' via *Hind*III. The plasmid used for this system, the induction procedure and the analysis are described elsewhere [38]. For cotransfection the *ble *gene of the plasmid pPhaT1 was substituted for the *nat1 *gene (provided by WERNER BioAgents, Jena, Germany) conferring resistance to nourseothricin. All constructs were checked for fidelity as well as number and orientation of integrated spacers by sequencing.

### Cell culture, transformation and in vivo localization

Cultivation and biolisitc transformation of *Phaeodactylum tricornutum *was carried out according to Apt et al. 1996 [39]. Cotransfected cells were selected on plates containing 75 μg/ml nourseothricin (WERNER BioAgents, Jena, Germany). Analysis of recombinant cells was performed with a Leica TCS SP2 confocal laser-scanning microscope using the settings as described [17] for the GFP and chlorophyll fluorescence. YFP images were obtained by excitation of the chromophore at 514 nm and the usage of a DD 458/514 beam splitter.

## Authors' contributions

GF carried out the experiments. MSS and UGM conceived the study. All authors analyzed the data and participated in writing, reading and approving the manuscript.

## Supplementary Material

Additional file 1 Additional table 1: PCR primers used in this study Click here for file
