# Supplementary material for: The physical and functional borders of transit peptide-like sequences in secondary endosymbionts
Source: BMC Plant Biol. 2010 Oct 19;10:223. doi: 10.1186/1471-2229-10-223 (PMC3017844; doi:10.1186/1471-2229-10-223)
Supplement: Additional file 1 — Additional table 1: PCR primers used in this study [file 1471-2229-10-223-S1.PDF]

Additional file 1

PCR primers used in this study

Direction	Description	Sequence (5´ → 3´)[a]
forward	FcpD_wt	gaattcatgaagactgctgtcattgc
reverse	FcpD_wt	aaccatggtggtggcaaccgaagtacgggccgcgttcttgg
forward	eGFP_wt	aaccatggtgagcaagggcgaggagc
reverse	eGFP_wt	ctaagcttacttgtacagctcgtccatgc
reverse	FcpD_K20E+R24E	aaccatggtggtggcaaccgaagtctcggccgcgttctcgg
reverse	FcpD_K20A+R24A	ccatggtggtggcaaccgaagtagcggccgcgttcgcggcagggg
forward	eGFP_K4E	aaccatggtgagcgagggcgaggagc
reverse	FcpD_K20R	aaccatggtggtggcaaccgaagtacgggccgcgttcctgg
forward	eGFP_K4R	aaccatggtgagcaggggcgaggagc
forward	eGFP_K4A	ccatggtgagcgcgggcgaggagc
reverse	FcpD_K20E+N21K+R24E	ttccatggtggtggcaaccgaagtctcggccgccttctc
reverse	FcpD_K20E+N21R+R24E	ttccatggtggtggcaaccgaagtctcggccgccctctc
reverse	FcpD_ΔK20-T30	ttccatggcaggggcaaaggcggcggc
forward	(self assembly) GFP11	agctaggagggtctggtgg
reverse	(self assembly) GFP11	aagcttggtaccttatgtaatcccagc

[a]Codons causing amino acid substitutions are underlined
